# Male partner involvement in efforts to eliminate mother-to-child transmission of HIV in Kisumu County, Western Kenya, 2015

**DOI:** 10.11604/pamj.supp.2017.28.1.9290

**Published:** 2017-11-04

**Authors:** Samuel Juma, Venny Nyambati, Mohamed Karama, Jane Githuku, Zeinab Gura

**Affiliations:** 1 Field Epidemiology and Laboratory Training Program (FELTP-K), Ministry of Health, Kenya; 2 Jomo Kenyatta University of Agriculture and Technology, Kenya; 3 Kenya Medical Research Institute (KEMRI), Kenya

**Keywords:** Caesarean section, indications, Kenya, vaginal birth, epidemiological study

## Abstract

**Introduction:**

rates of caesarean sections (CS) have been rising globally over time. Our study sought to identify factors associated with CS among mothers delivering at Mama Lucy Kibaki Hospital in Nairobi city county, Kenya.

**Methods:**

we conducted an unmatched case-control study using a 1:2 case-to-control ratio. Cases were defined as mothers who delivered through CS at the hospital while controls were defined as mothers who delivered through vaginal birth. Semi-structured questionnaires were used to interview study participants. We calculated means and proportions of demographic and obstetric characteristics of the mothers and crude odds ratios (cOR) and 95% confidence intervals (CI) to identify factors associated with CS. We performed multivariable logistic regression to identify independent factors associated with CS.

**Results:**

we enrolled 396 participants consisting of 132 cases and 264 controls. We identified 148 (56.1%) spontaneous vaginal births. There were 89 (67.4%) primary CS. Among the 67 (50.7%) cases that had intrapartum complications, 28 (41.8%) had arrest of labor while among the 52 (19.7%) controls that had intrapartum complications 21 (40.4%) had abnormal intra-partum bleeding. There were 85 (65.4%) emergency caesarean sections, out of which 33 (25%) were due to failure of labor to progress. Of the elective caesarean sections, 26 (19.7%) were repeat caesarean. Delivering a baby with a normal birth weight (cOR = 0.39,95% CI 0.18, 0.94) and being formally employed (cOR = 1.90 (95% CI 1.4, 3.1)) were associated with CS. Being formally employed (AOR = 1.78, p = 0.030) was independently associated with a mother undergoing CS compared to mothers who were not formally employed, while mothers whose babies had a normal birth weight, ≥ 2.5kg (AOR = 0.40, p = 0.040) were less likely to undergo CS as compared to mothers whose babies had a low birth weight.

**Conclusion:**

employment status and birth weight were associated with CS in urban Kenyan mothers.

## Introduction

Caesarean section rates are rising globally, but the determinants of this increase, especially in low-income and middle-income countries, are debatable. Since the late 70s there have been a steady increase in the proportion of women delivering through caesarean sections (CS). This led the World Health Organization (WHO) in 1985 to state that: "There is no justification for any region to have caesarean section rates higher than 10-15%" [1], since increased rates of CS do not give additional health benefits but may increase maternal risks, have implications for future pregnancies and put more strain on the available health resources [2]. Four decades later, however, the optimal rate of births by CS have remained relatively the same in both low income and high income countries [3].

The average CS rate in Africa was reported to be 9% by the WHO global survey on maternal and perinatal health and ranges between 0.6% to 18.0% [4]. Nationally and even Nairobi, the capital city and largest city in Kenya, CS rates have been increasing [5] and according to data available from the District Health Information Software (DHIS), the national CS rate was 14.4% while Nairobi county had a CS rate of 24.9% in 2014 (HMIS Kenya). The Nairobi Hospital, Kenyatta National Hospital and Aga Khan University Hospital had CS rates of 33%, 30% and 20.4% respectively [6].

Many factors are associated with CS such as mothers’ age, doctors’ preference, medical indications and mothers’ economic status [5]. Other indications for a CS include risk of uterine rupture, induction of labor, decreased use of operative vaginal delivery and other medical-legal concerns [7]. Maternal choice, increased safety for mother and baby and recognition of the pelvic damage associated with vaginal birth have also been associated with CS [8]. Whether medically indicated or self-requested, CS exerts pressure into the health care system because of the added cost involved and the possible complications associated with it [9]. High rates of CS are also known not to necessarily indicate better perinatal care and can be associated with harm [1]. Despite the mentioned risks there are reported benefits of CS which include greater safety for the baby, less pelvic floor trauma for the mother, avoidance of labor pain and convenience [10]. Although the proportions of CS births are documented, little is known about the factors associated with CS in Nairobi. We assessed the factors associated with CS at Mama Lucy Kibaki Hospital in Nairobi.

## Methods

### Study site and design study site and design

We conducted an unmatched case control study among mothers delivering at Mama Lucy Kibaki hospital between January and March, 2015. Mama Lucy Kibaki Hospital is a high volume government owned regional referral hospital in the eastern part of Nairobi Kenya’s capital city. In 2014 it conducted 6,645 vaginal births and 1,433 CS representing 7% of all the deliveries in Nairobi.

### Study population, inclusion criteria and case and control definitions

The study population constituted mothers aged 15-49 years who delivered at Mama Lucy Kibaki hospital. A case was defined as any caesarean birth conducted at the study site during the study period. A control was defined as any vaginal delivery either natural or assisted, conducted at the study site during the study period. We recruited into the study participants who delivered at the Mama Lucy Kibaki hospital, were willing and able to provide informed consent/assent for participation and met case and control definition.

### Sample size calculation and sampling procedure

A minimum sample size of 396 was calculated using Fleiss JL formula using a case: control ratio of 1:2, assuming a 95% confidence level, proportion of exposure of 21.4% and 80% power to detect odds ratio of 2. At the post-natal ward, patients who met the case definition were recruited to be in the study consecutively until the sample size was achieved. Systematic random sampling was used to recruit the controls whereby every third mother who met the definition of a control was recruited into the study. In instances when the third mother didn’t meet the criteria, she was replaced by the next consecutive mother that met the definition of a control.

### Data collection, management and analysis

We trained data collectors and pretested the questionnaire. Using the trained data collectors, we collected socio-demographic data and data on obstetric history of the participants, circumstances surrounding delivery, indications, parity, gravidity and baby’s birth weight using semi structured questionnaires administered to the mother post-delivery at the post-natal clinics. Additional information on mother’s presentation during pregnancy and the baby were extracted from the mother child booklet.

Descriptive analyses of the socio demographic and obstetric characteristics were done by calculating medians and proportions. For the bivariate analysis, the chi-square test was used to compare differences in various exposures of interest. We calculated odds ratios (OR) testing various exposures for associations (previous CS scar, antenatal clinic (ANC) attendance, employment status, birth weight, parity) with outcome variable (mode of delivery, either CS or vaginal). We included all exposures from the bivariate analysis with p-value < 0.15 into a logistic regression model using a forward selection process for the calculation of adjusted odds ratios (aOR), in which, all exposures with p-value < 0.05 were considered to be independently associated with caesarean section.

### Ethical consideration

Participation in the study was voluntary and participants were free to withdraw at any time. Written informed consent was obtained from the mothers who participated in the study. When underage mothers were encountered, assent was sought from them as well as an informed consent from an adult related to them. Confidentiality of the study participants was maintained by using questionnaires with unique identifiers to keep track of their names and keeping the collected data in a password protected database. All study procedures were reviewed and ethical clearance given by Jaramogi Oginga Odinga Teaching and Referral Hospital Ethics and Review Committee, ERC 1B/VOL.1/144.

## Results

### Socio-demographic factors

We enrolled 396 participants consisting of 132 cases and 264 controls. Characteristics of caesarean controls are described in Table 1.

**Table 1:** socio-demographic and obstetric characteristics of study cases and controls attending Mama Lucy Kibaki Hospital, Nairobi, Kenya, 2015

	Characteristics	Case # (%) N=132	Controls # (%) N= 264
Age in years	16	3 (2.3)	8 (3.0)
	17-25	61 (46.2)	133 (50.6)
	26-34	60 (45.5)	113 (43.0)
	35-49	8 (6.1)	9 (3.4)
Level of education attained	None	2 (1.5)	0 (0)
	Primary	29 (22)	65 (24.6)
	Secondary	64 (48.5)	115 (43.6)
	Tertiary	37 (28)	84 (31.8)
Marital status	Single	18 (13.6)	38 (14.5)
	Married	106 (80.3)	224 (85.5)
	Divorced/separated	8 (6.1)	0 (0)
Religion	Christianity	130 (99.2)	260 (98.9)
	Islam	1 (0.8)	3 (1.1)
Employment status	Employed	36 (27.3)	44 (16.7)
	Unemployed	96 (72.7)	220 (83.3)
Gravidity	Multigravida	75 (56.8)	159 (60.2)
	Primigravida	57 (43.2)	105 (39.8)
Parity	Multiparous	64 (49.5)	149 (56.3)
	Primiparous	68 (51.5)	115 (43.7)
ANC visits	Attended ANC	128 (97)	258 (98.5)
	Did not attend ANC	4 (3)	4 (1.5)
Frequency of ANC visits	≥ 4 visits	84 (65.6)	155 (60.1)
	˂ 4 visits	44 (34.4)	103 (39.9)
Family planning	Used family planning method	50 (37.8)	92 (35.6)
	Did not use family method	82 (62.2)	170 (64.4)
Birth Plan	Had birth plans	17 (12.2)	32 (12.2)
	Did not have birth plans	115 (87.7)	230 (87.8)

### Obstetric characteristics

Favorable pregnancy outcomes were reported among both cases and controls, with the proportion of live births being 97.7% (129/132) among cases and 100% (264/264) among controls. There were 127 (96.2%) singleton births among cases compared to 258 (98.1%) among controls. Among the cases, 64 (49.5%) mothers were multiparous while among the controls the mothers were 149 (56.3%). Primigravida mothers were 75 (56.8%) among cases and 159 (60.2%) among the controls. Among cases, 85 (65.4%) underwent emergency CS out of which 15 (11.3%) had postpartum complications and 16 (12.1%) had wound infection. Among controls, 40 (15.2%) had postpartum haemorrhage and 4 (1.5%) had pre-eclampsia. Among cases, intrapartum complications occurred in 28 (21.2%). Among the controls, intrapartum complications occurred in 21 (16%) mothers.

### Indications for CS

Medical indications for emergency CS included 28 (21.2%) mothers with foetal distress and 30 (22.7%) mothers with prolonged labor medical indications for the 47 (34.6%) women who delivered through elective CS included 26(19.7%) who had undergone a CS previously 13 (9.9%) who had Cephalo Pelvic Disproportion (CPD) and 2 (1.5%) with placenta praevia

At bivariate analysis, delivering a baby with a normal birth weight (cOR = 0.39, 95% CI 0.18, 0.94) and being formally employed (cOR = 1.90 95% CI 1.4, 3.1) were associated with mothers who had CS compared to mothers who had vaginal births (Table 2). After adjusting for age, parity and gravidity simultaneously, mothers undergoing CS had greater odds of being formally employed (AOR = 1.78, p = 0.030, 95% CI 1.06, 2.98) versus being unemployed compared to mothers who had vaginal birth, while mothers who had CS were at lower odds of delivering babies with normal birth weight, ≥ 2.5kg (AOR = 0.40, p = 0.040, 0.16, 0.96) versus being underweight, compared to mothers who had vaginal births (Table 3).

**Table 2:** bivariate analysis factors associated with mothers delivering at Mama Lucy Kibaki Hospital between January and March 2015

Factor	Variable	Cases N (%)	Controls N (%)	Crude OR (95% CI)	P Value
Birth weight	Normal birth weight	120 (95.1)	254 (97.7)	0.3 (0.7-0.94)	**0.04**
	Low birth weight	12 (4.9)	10 (2.3)	Ref.	
Employment status	Employed	82 (62.1)	120 (45.5)	1.97 (1.28-3.02)	**0.002**
	Not married	50 (37.9)	144 (54.5)	Ref.	
Education level	Secondary education and above	101 (76.6)	199 (75.4)	1.06 (0.65-1.74)	0.9
	Primary education and below	31 (23.4)	65 (24.6)	Ref.	
Gravidity	Multigravida	75 (56.8)	159 (60.2)	0.88 (0.57-1.33)	0.52
	Primigravida	57 (43.2)	105 (39.8)	Ref.	
Parity	Multiparous	64 (49.5)	149 (56.3)	0.73 (0.48-1.10)	0.14
	Primiparous	68 (51.5)	115 (43.7)	Ref.	
ANC visits	Attended ANC	128 (97)	258 (98.5)	0.5 (0.12-2.02)	0.45
	Did not attend ANC	4 (3)	4 (1.5)	Ref.	
Frequency of ANC visits	≥ 4 visits	84 (65.6)	155 (60.1)	1.27 (0.86-1.97)	0.32
	˂ 4 visits	44 (34.4)	103 (39.9)	Ref.	
Family planning	Used family planning method	50 (37.8)	92 (35.6)	1.12 (0.73-1.73)	0.68
	Did not use family method	82 (62.2)	170 (64.4)	Ref.	
Birth Plan	Had birth plans	17 (12.2)	32 (12.2)	1.06 (0.57-1.99)	0.87
	Did not have birth plans	115 (87.7)	230 (87.8)	Ref.	
Marital status	Married	106 (80.3)	224 (85.5)	0.69 (0.40-1.20)	0.2
	Not married	26 (16.7)	38 (14.5)	Ref.	
Birth outcomes	Twins	6 (4.9)	6 (2.3)	2.05 (0.65-6.48)	0.23
	Singletons	126 (95.1)	258 (97.7)	Ref.	

**Table 3:** factors independently associated with CS at Mama Lucy Kibaki Hospital between January and March 2015

	Characteristic	Caesarean Section (%)	Vaginal Delivery (%)	Adjusted OR (95% CI)
Birth weight	Normal (2.5-3.9kg)	120 (91.6)	254 (96.58	
	Low ((<2.5 Kg)	12 (8.4)	10 (3.42)	**0.40 (0.16, 0.96**
Employment status	Employed	82 (62.1)	120 (45.5)	
	Unemployed	50 (37.9)	144 (54.5)	**1.78 (1.06-2.98)**

## Discussion

Two factors were found to be associated with women who had CS in Nairobi, Kenya. Being employed was associated with a mother delivering through CS among women in Nairobi, Kenya. Studies in the United States and Brazil also found that employed women who were employed prenatally were more likely to deliver through CS compared to unemployed women [11,12]. We also found that mothers who underwent CS were at lower odds of having babies with normal birth weights because many of the low birth weight babies are premature and like babies with Cephalo pelvic disproportion are delivered through CS [13,14].

There were 66.7% of women with CS that attended a minimum of four antenatal visits, which was higher than the national average of 58% [15], but similar for the 60.1% of women with vaginal deliveries. This is because the national average includes both home deliveries as well as hospital deliveries whereas in this study only hospital deliveries are accounted for because it was hospital based.

Foetal distress and prolonged labor were most common among those with emergency CS. This is consistent with other studies carried out in the sub Saharan region that indicate that prolonged labor is the leading indication for CS followed by foetal distress [16-18]. Indications for elective CS reported were previous CS scar and Cephalo pelvic disproportion. This was also reported in the Agha Khan University Hospital, where a previous CS and absolute disproportion of maternal pelvis was associated with CS since they made vaginal delivery impossible [6]. This is also comparable to Nigeria, where the main indications for CS were failure of labor to progress, foetal indications, malpresentation and previous CS [19,20].

### Limitations

Our findings are only generalizable to women seeking delivering at the study hospital. However, as the proportion of hospital deliveries in the catchment population is high, then the findings provide reliable estimates for the catchment area.

## Conclusion

Maternal income and infant’s birth weight influenced the likelihood of delivering through caesarean section. To arrest the factors associated with CS the county department of health should promote focused ante natal care as this will help to identify pre-existing health conditions and detect early complications arising during the pregnancy.

### What is known about this topic

- The rates of caesarean sections have been on the rise despite the risks to the health of the mother and new-born;
- Besides the medical indications for caesarean section, there are external factors that influence the decision for surgical delivery.

### What this study adds

- The study highlights the factors associated with caesarean sections in a county referral hospital in Nairobi, Kenya and characterizes the obstetric and socio demographic features of the mothers delivering at this hospital.

## Competing interests

The authors declare no competing interest.
